# Hepatitis D virus infection prevalence in persons with human immunodeficiency virus and hepatitis B virus coinfection in Germany

**DOI:** 10.1007/s15010-025-02683-w

**Published:** 2025-11-07

**Authors:** Fauzi Elamouri, Thomas Lutz, Gabi Knecht, Christoph Wyen, Philip Posdzich, Malte Monin, Michael Sabranski, Christian Hoffmann, Alexander Killer, Björn Erik-Ole Jensen, Jakob J. Malin, Stefan Esser, Stefan Mauss, Roger Vogelmann, Christoph Boesecke, Daniel Beer, Stephan Grunwald, Annette Haberl, Florian Voit, Nazifa Qurishi, Sebastian Noe, Pavel Khaykin, Stephan M. Schneeweiß, Stefan Christensen, Carolynne Schwarze-Zander, Arne Jessen, Anders Boyd, Jürgen K. Rockstroh, Kathrin van Bremen

**Affiliations:** 1 Department of Medicine I, University Hospital Bonn, Venusberg Campus 1, Building 26, 53127 Bonn, Germany; 2 Medical School Hamburg, Hamburg, Germany; 3 Infektiologikum Frankfurt, Frankfurt, Germany; 4 Praxis am Ebertplatz, Köln, Germany; 5 Division of Infectious Diseases, Department I of Internal Medicine, University Hospital of Cologne, Cologne, Germany; 6 ICH, Hamburg, Germany; 7 Department of Oncology, Haematology and Bone Marrow Transplantation with Section Pneumology, University Medical Center Hamburg-Eppendorf, Hamburg, Germany; 8 Department for Gastroenterology, Hepatology and Infectious Diseases, Medical Faculty, University Hospital Düsseldorf, Heinrich Heine University Düsseldorf, Düsseldorf, Germany; 9 Clinic of Dermatology, Department of Venerology, University Hospital Essen, Essen, Germany; 10 MVZ Düsseldorf, Düsseldorf, Germany; 11 Mannheimer Onkologie Praxis, Mannheim, Germany; 12 Praxiszentrum Blondelstrasse, Aachen, Germany; 13 Zentrum für Infektiologie, Berlin, Germany; 14 Goethe-University Hospital, Medical Clinic 2, Infectious Diseases, Frankfurt, Germany; 15 TUM School of Medicine and Health, Clinical Department of Internal Medicine II, TUM University Hospital, Munich, Germany; 16 Praxis Gotenring, Köln, Germany; 17 MVZ München am Goetheplatz, München, Germany; 18 MainFachArzt, Frankfurt, Germany; 19 Praxis Hohenstaufenring, Köln, Germany; 20 Centrum für Infektiologie, Münster, Germany; 21 Department for Internal Medicine B, Gastroenterology, Hepatology, Infectious Diseases, University Hospital Muenster, Muenster, Germany; 22 Gemeinschaftspraxis am Kaiserplatz, Bonn, Germany; 23 Praxis Jessen, Berlin, Germany; 24 Stichting HIV Monitoring, Amsterdam, Netherlands; 25 MVZ Innere Medizin, Köln, Germany

**Keywords:** HIV, HBV. hepatitis delta, Screening

## Abstract

**Purpose:**

People with HIV (PWH) who have chronic hepatitis B virus (HBV) coinfection are at increased risk of also having hepatitis D virus (HDV) infection given the shared transmission pathways. The current prevalence of HDV in Germany among people with HIV/HBV, however, is unknown. The aim of this study was to determine the percent with HDV screening as well as the current HDV prevalence among German PWH with HBV coinfection and underlying risk factors for HDV infection.

**Methods:**

21 German HIV treatment centers (6 university clinics, 15 private practices) recruited all people with a confirmed HIV diagnosis and a positive hepatitis B surface antigen for more than 6 months, aged ≥ 18 years, and actively in care on December 31, 2023. We assessed the percent with anti-HDV antibody testing in the total cohort. In addition, we calculated the prevalence of individuals who ever had an anti-HDV positive serology (i.e., past/current infection) and the prevalence of individuals whose last HDV RNA result was positive (i.e., active infection).

**Results:**

Overall, 458 PWH with HBV coinfection were included in the analysis. 17% of the participants were female and 83% male. Median age was 55 years (IQR 48–61). 99% of participants were receiving antiviral dual active therapy with 84% having undetectable HIV viral load and 90.8% having undetectable HBV-DNA. Anti-HDV antibody results were available in 370 (81%). Of these, 27 (7.3%) had a previous/current HDV infection. HDV RNA testing was performed in 24/27 participants with HDV-positive serology, of whom 14/24 (58%) were positive.

**Conclusion:**

In Germany, 7% of PWH with HBV coinfection who underwent HDV screening had HDV antibodies with only half showing signs of active HDV replication.

## Introduction

Hepatitis D is caused by a defective RNA virus, considered one of the smallest known human pathogens. HDV requires the presence of HBsAg to enter hepatocytes and replicate [1, 2]. Consequently, HDV can only be acquired through coinfection with HBV or through superinfection in individuals already chronically infected with HBV [3]. Acute HDV-HBV coinfection is followed by clearance of both viruses in approximately 80–95% of people [1]. In contrast HDV superinfection in an individual with HBV results in chronic HDV-HBV infection in more than 70–90% of cases [2, 4]. Persistent HDV infection is associated with accelerated liver fibrosis, often culminating in cirrhosis and a substantially increased risk of hepatocellular carcinoma (HCC) [5, 6]. Although the widespread availability of effective HBV vaccination has markedly reduced HDV incidence in high-income countries, the global burden remains considerable. Current estimates suggest that between 12 and 72 million individuals worldwide are living with chronic HDV infection [4, 7, 8].

In the subgroup of PWH, who are at increased risk for acquiring viral hepatitis via shared transmission pathways (including sexual contact, blood transfusion, and injection drug use). the prevalence of HDV infection has been reported between 10% and 20% of persons with HIV/HBV, with variations according to region as well as transmission groups [9, 10]. Particularly, early in the HIV epidemic, higher rates of HDV have been reported for persons with substance use disorder [9]. More recently, a shift from people who acquired HIV through substance use to men who have sex with men (MSM) has been reported with HDV prevalence of around 6.3% [11]. Common for all PWH with HBV/HDV-coinfection is a more rapid progression of liver disease [12] resulting in increased rates of decompensated cirrhosis and higher liver disease-related mortality compared to those without HIV [13, 14]. Therefore, mandatory screening for HDV in persons with HIV/HBV was implemented into the current European AIDS Clinical Society (EACS) guideline recommendations [15]. However, there is still a lack of testing in daily routine, even at sites where testing is easily accessible and reimbursed by healthcare providers. Moreover, most PWH who are coinfected with HBV are tested at initial HBV diagnosis, but after commencing antiretroviral treatment, which usually includes a tenofovir-based therapy, follow-up testing of HDV does not routinely take place.

The aim of this study was to assess the implementation of EACS guidelines for anti-HDV antibody-testing among PWH from Germany with positive Hepatitis-B-Surface-Antigen (HBsAg). Additionally, we also intended to evaluate the prevalence of HDV infection among individuals with HIV/HBV-coinfected individuals in 2023, as well as the corresponding risk factors for HDV-coinfection.

## Methods

### Study design

This is a secondary analysis of German data from the HDV-Europe study. In brief, the HDV-Europe study was a cross-sectional, multi-country cohort aimed to evaluate the HDV testing uptake and HDV infection prevalence in individuals with HIV and HBV from Italy, Spain, Germany, the Netherlands, Switzerland, Poland and the United Kingdom. The research question of the primary analysis was to compare HDV testing rates and HDV prevalence between different European regions. Data were either retrieved from pre-existing cohorts or collected from outpatient clinical centers. The inclusion criteria were as follows: 18 years of age or older, confirmed HIV-1 infection (with HIV RNA PCR or western blot), documentation of two positive HBsAg results > 6 months apart, and the last clinical visit occurring between 1 January 2023 and 31 December 2023.

The study was approved by Bonn University medical ethics committee (reference number 352/23-EP) and the coordinating centers of each participating country. Consent for use of data was obtained in accordance with the Declaration of Helsinki and data transfer respected the European Union’s General Data Protection Regulations.

In this study, we included only individuals from the HDV-Europe study who were recruited in Germany, had completed data entry, and had information on HDV testing status. The aim of this secondary analysis was to better understand HDV prevalence in Germany among PWH and to assess possible risk factors for HDV transmission. Moreover, the liver disease stage was analyzed for those individuals with concomitant HDV infection.

### Study variables

Data were entered in an electronic clinical report form by study clinicians/nurses. Data entry was facilitated using RedCap and data were stored on servers located at the Fundación SEIMC-GESIDA (Madrid, Spain). For this study, we used data extracted on 26 June 2025.

We collected data on the following basic demographic variables: sex at birth, date of birth, ethnicity, country of birth and year of migration where applicable. We collected data on alcohol consumption (dichotomized into up to two or three standard drinks per day or more for women and men, respectively). Data retrieved on HIV infection were as follows: mode of HIV transmission, date of HIV diagnosis, any AIDS-defining illnesses, most recent CD4 + T-cell count and most recent HIV RNA result (i.e., detectable or undetectable using a commercially available PCR assay). Data on HBV infection included the following: date of first positive HBsAg test, HBV DNA measurement at entry into care, most recent quantitative HBsAg result, hepatitis B “e” antigen (HBeAg) status, and HBV RNA result (i.e., detectable or undetectable using a commercially available PCR assay). We also asked if the participant had commenced an anti-HBV treatment and, if so, which antiviral agent(s) were used. Data on hepatitis C virus (HCV) infection were also collected and included anti-HCV antibody and HCV RNA results.

Data on HDV infection included the following: whether HDV serology had been performed and if so, the result of the anti-HDV test, whether an HDV RNA level was measured and if so, both the qualitative (i.e., detectable or undetectable) and quantitative result. We asked if the participant had commenced an anti-HDV therapy and whether this therapy was currently ongoing (i.e., pegylated interferon-α or bulevirtide).

Data on liver disease included the following: prior diagnosis of liver cirrhosis (including method used to diagnose liver cirrhosis), transient elastography measurements, previous history of decompensated liver disease, HCC, or liver transplantation.

### Statistical analysis

Data were described using counts and proportions for categorical variables and medians and interquartile ranges (IQR) for continuous variables. We compared the distributions of individuals tested for HDV versus not tested and anti-HDV positive versus negative using Pearson’s χ^2^ test or Fisher’s Exact test for categorical variables and Kruskal-Wallis test for continuous variables. Analysis was carried out using Stata/SE (v17.0, College Station, TX, USA). Significance was determined using a *p*-value < 0.05.

## Results

### Description of the study population

**Fig. 1:**
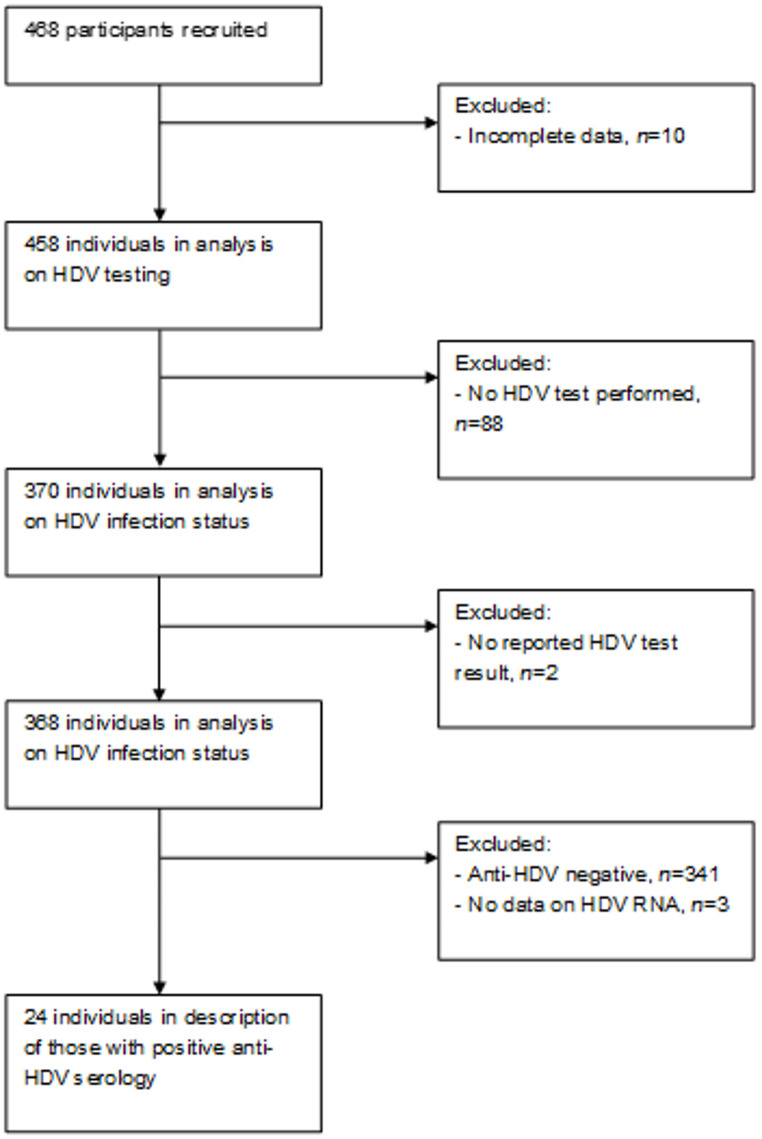
Participant flow of the included individuals with HIV and HBV in Germany

Participants were identified from 21 clinics across Germany (6 University clinics and 15 private practices). Initially, 468 individuals with HIV and HBV were identified as eligible for the study. Of them, 458 had complete data entered and were considered for subsequent analyses (Fig. 1).

Individuals were predominantly male at birth (*n* = 380; 83.0%) and 219 (48.1%) were born in Germany. Two-thirds were men who had sex with men and the remainder mainly had acquired HIV through heterosexual or other contact (30.3%) and a few were persons with substance use (4.4%). As the median of the most recent CD4 cell count was 631 cells/mm^3^ (IQR = 445–874) and the most recent HIV RNA viral load was undetectable in 84.1% of individuals.

The median time from the first HBsAg-positive test was 16 years (IQR = 9–22). At the most recent measurement, HBsAg levels were a median 3.03 log_10_ IU/mL (IQR = 3.01–3.64), roughly one-quarter were HBeAg-positive and HBV DNA was undetectable in 90.8%. The vast majority (98.9%) were undergoing an anti-HBV therapy and had commenced their first anti-HBV therapy a median of 16 years before data extraction.

### HDV testing in individuals with HIV and HBV

Of the 458 included participants, 370 (80.7%) had been tested at least once for HDV via antibody testing. The characteristics of individuals with HIV and HBV are summarized for those tested and not tested for anti-HDV antibodies in Table 1. There were no noticeable differences in characteristics between those tested versus not tested; however, in the tested persons the percentage with excessive alcohol consumption, HBeAg-positive serology, and percentage with undetectable HBV DNA was higher than in those who had not been tested (*p* = 0.026 and 0.043, respectively).

**Table 1:** Characteristics of the study population of individuals with HIV and HBV coinfection in Germany, stratified on hepatitis D virus (HDV) testing status

Characteristics	Total	HDV-testing status		*p*-value*
		Not tested	Tested	
	(*n* = 458)	(*n* = 88)	(*n* = 370)	
Age, years	56 (48–62)	53 (48–62)	56 (48–62)	0.82
Male sex at birth	380 (83.0)	74 (84.1)	306 (82.7)	0.89
Missing	8	1	7	
Born in Germany	219 (48.1)	47 (53.0)	172 (46.7)	0.22
Missing	3	1	2	
WHO region of origin				0.29
Western Europe	249 (54.4)	56 (63.6)	193 (52.2)	
Eastern Europe	15 (3.3)	1 (1.4)	14 (3.8)	
Africa	94 (20.5)	11 (12.5)	83 (22.4)	
South-East Asia	17 (3.7)	4 (4.6)	13 (3.5)	
Other	73 (15.9)	14 (15.9)	59 (16.0)	
Missing	3	1	2	
Excessive alcohol consumption	23 (6.8)	10 (13.2)	13 (5.0)	0.012
Missing	120	12	108	
Mode of HIV transmission				0.26
MSM	255 (65.4)	53 (66.3)	202 (65.2)	
PWID	17 (4.4)	5 (6.3)	12 (3.9)	
Heterosexual/other	118 (30.3)	22 (27.5)	96 (31.0)	
Missing	68	8	60	
Years since first HIV positive test	21 (13–29)	21 (15–28)	21 (13–29)	0.71
Missing	27	7	20	
Ever an AIDS-defining illness	147 (35.2)	30 (35.7)	117 (35.0)	0.91
Missing	40	4	36	
CD4 + T cell count^†^, per mm^3^	631 (445–874)	639 (448–872)	629 (445–874)	0.85
Missing	3	0	3	
Undetectable HIV RNA^†^	376 (84.1)	77 (87.5)	299 (83.3)	0.33
Missing	11	0	11	
Years since first HBsAg positive test	16 (9–22)	17 (9–23)	13 (9–22)	0.64
Missing	153	21	132	
HBsAg levels^†^, log_10_ IU/mL	3.03 (3.01–3.64)	2.97 (1.86–3.56)	3.05 (2.16–3.65)	0.39
Missing	169	53	169	
HBeAg positive	112 (24.4)	25 (28.4)	87 (23.5)	0.026
Missing	137	34	103	
Receiving anti-HBV therapy	453 (98.9)	87 (98.9)	366 (98.9)	0.96
Any TDF/TAF use^‡^	390 (86.1)	80 (92.0)	310 (84.7)	0.079
Any XTC use^‡^	287 (63.4)	58 (66.7)	229 (62.6)	0.48
Years since commencing anti-HBV therapy^‡^	16 (9–22)	16 (9–21)	15 (9–21)	0.60
Missing	122	23	99	
Undetectable HBV DNA^†#^	416 (90.8)	75 (85.2)	341 (92.2)	0.043
Anti-HCV positive	43 (9.4)	12 (13.6)	31 (8.4)	0.16
Missing	13	4	9	
Detectable HCV RNA^†¶^	7 (16.3)	1 (8.3)	6 (19.4)	0.66
Missing	2	0	2	
ALT 2xULN	24 (5.2)	4 (4.6)	20 (5.4)	0.75
ALT 5xULN	3 (0.7)	1 (1.1)	2 (0.5)	0.53
Type of clinic				0.71
University clinic	347 (75.8)	68 (77.3)	279 (75.4)	
Non-university clinic	111 (24.2)	20 (22.7)	91 (24.6)	

All data reported are *n* (%) or median (interquartile range), unless otherwise specified. Missing data are not included in the calculation of %

*Distribution of characteristics compared between HDV-testing groups using Kruskal-Wallis test for continuous variables and Pearson’s *χ*^2^ test or Fisher’s Exact test for categorical variables

^†^Most recent measurement

^‡^Only among individuals who are receiving anti-HBV therapy

^#^assuming the 398 missing HBV DNA viral loads were undetectable

^¶^Only among individuals with an anti-HCV positive serological test

Abbreviations: AIDS, acquired immunodeficiency syndrome; HBV, hepatitis B virus; HBeAg, hepatitis B “e” antigen; HBsAg, hepatitis B surface antigen; HCV, hepatitis C virus; HDV, hepatitis D virus; HIV, human immunodeficiency virus; MSM, men who has sex with men; PWID, person who injects(-ed) drugs; TAF, tenofovir alafenamide; TDF, tenofovir disoproxil fumarate; ULN, upper limit of normal; WHO, World Health Organization; XTC, lamivudine or emtricitabine. WHO, World Health Organization

### HDV positivity in individuals with HIV and HBV

Of the 370 who had been tested at least once for HDV, 368 (99.5%) had an available HDV serological result. Of them, 27 (7.3%) tested positive for anti-HDV antibodies. The characteristics of individuals with HIV and HBV are summarized for those testing positive and negative for anti-HDV antibodies in Table 2. Those testing positive for HDV were more often born in Eastern Europe (*p* = 0.002), and were less likely to be MSM and more likely to be PWID (*p* < 0.001) compared to those testing negative. In addition, those testing positive for HDV were more commonly anti-HCV antibody positive (*p* < 0.001) and had elevated ALT levels (i.e., 2x or 5x higher than the upper limit of normal, that is, 35 IU/mL; *p* = 0.019 and *p* < 0.001, respectively) than those testing negative (*p* < 0.001).

**Table 2:** Characteristics of the study population of individuals with HIV, HBV and HDV in Germany, stratified on HDV infection status

Characteristics	Total	HDV status		*p*-value*
		Negative	Positive	
	(*n* = 368)	(*n* = 341)	(*n* = 27)	
Age, years	56 (48–62)	56 (48–62)	52 (46–58)	0.052
Male sex at birth	304 (84.2)	282 (84.4)	22 (81.5)	0.69
Missing	7	7	0	
Born in Germany	172 (47.0)	166 (49.0)	6 (22.2)	0.007
Missing	2	2	0	
WHO region of origin				0.002
Western Europe	192 (52.5)	181 (53.4)	11 (40.7)	
Eastern Europe	14 (3.8)	9 (2.7)	5 (18.5)	
Africa	82 (22.4)	76 (22.4)	6 (22.2)	
South-East Asia	13 (3.6)	12 (3.5)	1 (3.7)	
Other	59 (16.1)	56 (16.2)	3 (11.1)	
Missing	2	0	2	
Excessive alcohol consumption	13 (5.0)	11 (4.5)	2 (12.5)	0.16
Missing	108	997	11	
Mode of HIV transmission				< 0.001
MSM	201 (65.3)	192 (66.9)	9 (42.9)	
PWID	12 (3.9)	6 (2.1)	6 (28.6)	
Heterosexual/other	95 (30.8)	89 (31.0)	6 (28.6)	
Missing	60	54	6	
Years since first HIV positive test	21 (13–29)	22 (13–29)	21 (14–26)	0.56
Missing	20	17	3	
Ever an AIDS-defining illness	116 (34.9)	108 (35.0)	8 (34.8)	0.99
Missing	36	32	4	
CD4 + T cell count^†^, per mm^3^	630 (448–874)	642 (459–880)	610 (378–872)	0.27
Missing	3	3	0	
Undetectable HIV RNA^†^	297 (83.2)	275 (83.1)	22 (84.6)	0.84
Missing	11	10	1	
Years since first HBsAg positive test	16 (9–21)	16 (9–22)	15 (9–21)	0.87
Missing	130	121	9	
HBsAg levels^†^, log_10_ IU/mL	3.05 (2.16–3.65)	3.03 (2.09–3.63)	3.42 (2.16–3.65)	0.26
Missing	167	153	14	
HBeAg positive	86 (32.3)	79 (31.9)	7 (38.9)	0.54
Missing	102	93	9	
Receiving anti-HBV therapy	364 (98.9)	337 (98.8)	27 (100=)	0.99
Any TDF/TAF use^‡^	308 (84.6)	284 (84.3)	24 (88.9)	0.52
Any XTC use^‡^	228 (62.6)	211 (62.6)	17 (63.0)	0.97
Any entecavir use^‡^	9 (2.5)	9 (2.7)	0	0.39
Years since commencing anti-HBV therapy^‡^	15 (9–21)	16 (9–22)	13 (8–20)	0.32
Missing	97	88	9	
Undetectable HBV DNA^†#^	339 (92.1)	313 (91.8)	26 (96.3)	0.40
Anti-HCV positive	31 (8.4)	20 (5.9)	11 (40.7)	< 0.001
Missing	8	8	0	
Detectable HCV RNA^†¶^	6 (19.4)	6 (30.0)	0 (0)	0.066
ALT 2xULN	19 (5.2)	15 (4.4)	4 (14.8)	0.019
ALT 5xULN	2 (0.5)	0 (0)	2 (7.4)	< 0.001
Type of clinic				0.75
University clinic	277 (75.3)	256 (75.1)	21 (77.8)	
Non-university clinic	91 (24.7)	85 (24.9)	6 (22.2)	

All data reported are *n* (%) or median (interquartile range), unless otherwise specified. Missing data are not included in the calculation of %

*Two individuals were excluded from this analysis as they were tested for anti-HDV antibodies, but did not have a test result

*Distribution of characteristics compared between active HDV status groups using Kruskal-Wallis test for continuous variables and Pearson’s *χ*^2^ test or Fisher’s Exact test for categorical variables

^†^Most recent measurement

^‡^Only among individuals who are receiving anti-HBV therapy

^#^Assuming the 325 missing HBV DNA viral loads were undetectable

^¶^Only among individuals with an anti-HCV positive serological test

Abbreviations: AIDS, acquired immunodeficiency syndrome; HBV, hepatitis B virus; HBeAg, hepatitis B “e” antigen; HBsAg, hepatitis B surface antigen; HCV, hepatitis C virus; HDV, hepatitis D virus; HIV, human immunodeficiency virus; MSM, men who has sex with men; PWID, person who injects(-ed) drugs; TAF, tenofovir alafenamide; TDF, tenofovir disoproxil fumarate; ULN, upper limit of normal; WHO, World Health Organization; XTC, lamivudine or emtricitabine

### Clinical outcomes of individuals with HIV, HBV and active HDV infection

Of the 27 individuals who were anti-HDV antibody positive, 24 (88.9%) had an available HDV RNA result. Of them, 14 (58.3%) had detectable HDV RNA and hence had active HDV infection. When considering the original population tested for anti-HDV antibodies, active HDV infection was observed in 3.8% (*n* = 14/370). For the individuals with active infection, 6 (43%) had previous/ongoing anti-HDV therapy and as of the last clinical visit, three were undergoing therapy with bulevirtide monotherapy. The distribution of liver diseases for individuals with past or active HCV infection is described in Table 3. The prevalence of all liver diseases was low, with liver cirrhosis being the most common (20% in those with past and 14% in those with active infection). Two individuals had a history of decompensated liver disease, both diagnosed by clinically detectable ascites. One individual was diagnosed with HCC and one received a liver transplantation.

**Table 3:** Clinical description of individuals with HIV, HBV and positive HDV infection

Clinical outcome	HDV infection status	
	Past infection	Active infection
	(*n* = 10)	(*n* = 14)
Current/prior diagnosis of liver cirrhosis*	2 (20)	2 (14)
Previous history of decompensated liver disease	1 (10)	1 (7)
Ever diagnosed with HCC	1 (10)	0
Received liver transplantation	1 (10)	0

All data reported are *n* (%)

*Information used to assess diagnosis of liver cirrhosis (sources are not mutually exclusive): liver biopsy (*n* = 1), non-invasive measurement (*n* = 2), clinical/biological (*n* = 3)

Abbreviations: HCC, hepatocellular carcinoma; HDV, hepatitis D virus

## Discussion

Globally, an estimated 5–6% of PWH also have HBV [16]. Within this subgroup, the reported prevalence of HDV among individuals with HIV, HBV, and HDV varies between 4% and 15% [9, 10, 17]. The highest HDV rates —approaching 50%— have been observed among PWH who injected drugs [9].

Within this substudy of the larger European HDV study including Italy, Spain, Germany, the Netherlands, Switzerland, Poland and the United Kingdom we assessed both the percent screening for HDV and prevalence for HDV in individuals with HIV/HBV in Germany and looked at HDV transmission risk factors. Overall, only 7.3% had detectable anti-HDV antibodies, and among them, 58% showed evidence of active HDV replication. It is important to emphasize that 88% of persons with positive HDV-serology were tested for HDV RNA, reassuring that there would be very little missed active replicating HDV diagnoses. This relatively low HDV prevalence contrasts sharply with the higher prevalence reported by EuroSIDA and other national European HIV cohorts from Italy and France [9, 10, 18, 19]. Differences in the distribution of key populations may partly explain the lower prevalence in Germany. The highest HDV prevalence is generally found among current or former people with substance use, a population that remains more common in Eastern Europe and was historically more prevalent in Spain and Italy during the early HIV epidemic [20, 21]. In contrast, Germany has a comparatively smaller population of persons with substance use living with HIV. Additionally, many persons with substance use in Western European cohorts represent individuals from the early HIV epidemic, a large proportion of whom have since died from complications of chronic liver disease. Indeed, because of the faster progression of liver fibrosis in PWH with HIV/HBV, liver disease related complications (hepatic decompensation and/or HCC) occur faster and at a much higher rate than in persons without HIV [13, 14]. The potential impact of other potentially contributing liver disease etiologies such as alcoholic liver disease and MASH also need to be discussed in this context.

Risk factors for testing positive for HDV in our national cohort were being born in Eastern Europe, and more likely to be PWID compared to those testing negative. In addition, those testing positive for HDV were more commonly anti-HCV antibody positive and had elevated ALT levels. Similar t data was recently published from a sample of adults with HIV/HBV in care in the US, of whom 4.0% were HDV IgG antibody-positive, among whom 41.7% had detectable HDV RNA [17]. History of substance use was again associated with exposure to HDV infection similarly to our study. These findings emphasize the importance of HDV testing among persons with HIV/HBV coinfection, especially those with a history of substance use [17]. Clearly, reflex testing for HDV in PWH and chronic hepatitis B needs to be implemented in clinical practice.

Interestingly, HDV is rarely detected in MSM which is by far the largest group of persons living with HIV included in our study. However, a recent study from Taiwan reported at baseline a HDV infection in 6.3% of individuals with HIV/HBV [11]. 87% of whom were MSM, suggesting that the transmission dynamics of HDV may be shifting [11]. Indeed, during 3987.78 person-years of follow-up (PYFU), 50 (10.0%) of 498 anti–HDV-negative PWH seroconverted HDV-positive, with an overall incidence rate of 12.54 per 1000 PYFU; 88.0% (44/50) of the HDV seroconverters were men who have sex with men [11]. Although, these high HDV seroconversion rates have not yet been reported for MSM groups from other countries this finding does underline that HDV screening is essential for all key populations. Moreover, it should be noted that in our German cohort there were 9 MSM with HDV antibodies, further supporting the need for HDV screening in this key population.

The wide variation in reported HDV prevalence is also partially explained by a lack of recent, reliable prevalence data; limited testing coverage, particularly in key and vulnerable populations; and substantial regional heterogeneity in HDV epidemiology. Notably, there is no consistent correlation between the geographic prevalence of HDV and HBV.

Of note, individuals with excessive alcohol intake were more likely to be tested for HDV indicating that in general there may be a tendency for increased testing in those persons with additional risk factors for progressive liver disease or persistently elevated liver enzymes because of other liver disease etiologies. Unfortunately, no information on further liver disease such as MASH were collected in this study.

Despite the recommendation of in the European Association for the Study of the Liver (EASL) and EACS guidelines to perform HDV antibody screening in all individuals with chronic HBV infection, HDV antibody results were only available for 81% of participants in the German cohort. This underscores a persistent gap between guideline recommendations and their implementation in clinical practice. Similar findings have been reported from several studies from the United States and Europe, which consistently describe low proportion of HDV screening among PWH with HBV [19, 20, 22, 23]. For example, the EACS hepatitis audit conducted in Georgia, Germany, Poland, Spain, and Romania found that HDV testing was performed in fewer than 50% of PWH with HBV [23].

Given the more aggressive progression of liver disease in PLWH, characterized by higher rates of cirrhosis, HCC, and liver-related mortality, timely diagnosis of HDV infection is of critical importance to enable HDV-specific therapy [12, 13]. Notably, coinfection with HIV, HBV, and HDV has been associated with a 6–9-fold higher risk of HCC compared to HIV/HBV coinfection [12]. In light of the availability of effective HDV therapies, improving early detection in PLWH is essential to prevent advanced liver disease and its complications. In our small group of individuals with HBV/HDV coinfection, advanced liver disease was also common, again confirming the faster course of liver disease and associated complications in this group.

It should also be emphasized that the determination of the current number of active HDV cases on a national level is helpful in modelling expected HDV treatment costs, considering the high treatment costs associated with modern HDV therapy. The much lower HDV prevalence described in this study at least promises lower expenses on HDV treatment than modelled from the large European cohort data with a prevalence of 15% [10].

Our study does have several limitations. In particular the cross-sectional nature of the dataset and the lack of longitudinal data/follow-up needs to be mentioned. In addition, the study population was mainly composed of MSM and men. Although the distribution of key populations in our study might differ from others in Europe, the individuals included do still represent the general epidemiology of HIV in Germany. Also, the important key population of prisoners was not addressed in this study. Furthermore, viral loads for HIV were captured as detectable or undetectable and only at the last clinical visit, which does not allow for providing exact virus levels or to discriminate between those with transient HIV versus real virological failure. It is therefore difficult to assess why almost 15% had detectable HIV RNA levels despite being on effective antiretroviral therapy. Finally, there were substantial missing data for some of the variables, notably HBV DNA viral loads at the last clinical measurement. There is a tendency for some clinical centers not to monitor HBV DNA levels if a person is undergoing tenofovir-containing antiretroviral therapy with undetectable HIV RNA [24]. For this reason, we assumed that missing represented undetectable HBV DNA levels in analysis.

## Conclusions

In Germany, approximately one in thirteen individuals with HIV/HBV coinfection who are actively in care show evidence of past or current HDV infection. Less than half of these exhibit ongoing HDV replication and therefore require evaluation for HDV therapy. HDV screening was conducted in accordance with guideline recommendations in 80% of cases, highlighting that further improvement in HDV screening practices is still needed. Reflex testing for HDV coinfection in hepatitis B surface antigen (HBsAg)-positive patients should be preferred over risk factor–based screening to avoid missed HDV diagnoses. With the advent of more effective HDV treatment options, timely HDV diagnosis has become increasingly important for potential HDV control and eradication, as well as for improving clinical outcomes in HDV-associated liver disease. The markedly faster progression of liver disease in HIV/HBV/HDV coinfected individuals underscores that successful suppression of HDV replication—and ultimately eradication under modern HDV therapies—can improve survival and liver outcomes. These benefits make new HDV treatments highly likely to be cost-effective.

## Data Availability

The data that support the findings of this study are not openly available due to reasons of sensitivity and are available from the corresponding author upon reasonable request. Data are located in controlled access data storage in RedCap.
